# Physiological and Molecular Mechanisms of Methionine Restriction

**DOI:** 10.3389/fendo.2018.00217

**Published:** 2018-05-04

**Authors:** Mary Neslund Latimer, Khalid Walid Freij, Beth M. Cleveland, Peggy R. Biga

**Affiliations:** ^1^Department of Biology, University of Alabama at Birmingham, Birmingham, AL, United States; ^2^National Center for Cool and Cold Water Aquaculture, Agricultural Research Service (USDA), Kearneysville, WV, United States

**Keywords:** microRNA, methionine restriction, stress, physiological, caloric restriction, mechanisms

## Abstract

Methionine restriction (MR) has been studied extensively over the last 25 years for its role in altering metabolic hallmarks of disease. Animals subjected to MR, display changes in metabolic flexibility demonstrated by increases in energy expenditure, glucose tolerance, and lifespan. These changes have been well characterized in a number of model systems and significant progress has been made in understanding how hepatic fibroblast growth factor 21 links MR to several components of its metabolic phenotype. Despite these advances, a complete understanding of mechanisms engaged by dietary MR remains elusive. In this review, we offer a brief history of MR and its known mechanisms associated with stress, metabolism, and lifespan extension. We consider the role of epigenetics in the response of animals to MR and propose a novel epigenetic pathway involving the regulation of microRNAs during MR.

## Introduction

Methionine restriction (MR) is known to produce lifespan extension and has been more recently investigated for its role in improving metabolic health. While much is known about the resulting phenotype following MR, a complete picture of mechanisms involved in MRs metabolic phenotype is less clear. At a molecular level, methionine metabolism creates *S*-adenosylmethionine (SAM), which acts as a master methyl donor. Methylation of cytosines in promoter regions of genes can regulate gene expression by controlling binding of transcription factors (1). Changes in methionine availability through dietary MR have the potential to decrease these available methyl donors and thus change transcriptional status of certain genes. One of these potential genes is fibroblast growth factor 21 (FGF21) which has been implicated in the metabolic responses to MR (i.e., increased energy intake and expenditure as well as increased insulin sensitivity) (2, 3). While increases in circulating and hepatic FGF21 are responsible for certain aspects of MRs metabolic phenotype, FGF21^−/−^ mice did not exhibit changes in transcriptional responses to MR in the liver (2). This FGF21 independent mechanism could potentially be linked to epigenetic changes that take place during MR in the liver when there is a reduction in methyl donors.

In this review, we explore the mechanisms of MR that could drive these epigenetic responses. One possible mechanism that has emerged as a factor in MR phenotypes is microRNAs (miRNAs). Studies have shown that MR alters miRNA expression levels in rainbow trout myosatellite cells (4, 5) and bone structures of mice (6), supporting a proposed connection between MR and miRNAs. This review will discuss the history and phenotypes associated with MR, as well as the role for miRNAs in MR phenotypes.

## Methionine Restriction

Dietary interventions have been studied for many decades in fields from nutrition to healthy aging. The most investigated intervention has been caloric restriction (CR), which has been shown to increase lifespan and health span (7). While CR, defined as a decrease of 30–60% from *ad libitum* feeding, is a well-known and effective treatment, first demonstrated in 1935 (8), compliance is often low. Alternatives to CR such as protein restriction (*de novo* amino acid restriction) (9, 10) have been used in patients with renal failure and more recently in mice as a lifespan extension strategy (11). Following studies on protein restriction, Segall et al. began to explore the effects of long-term tryptophan restriction in rats (12–14). While this proved to be a lifespan extension strategy hypothesized to work by decreasing levels of serotonin in the brain, early mortality of animals was a significant problem and led to abandonment of this strategy (15, 16). Orentreich et al. were the first to propose MR as an alternative therapy to increase lifespan (17). Early on, it was established that the effects seen during MR were not due to *de novo* CR, but rather some other mechanism. Orentreich et al. pair-fed control diets based on amounts consumed by rats receiving MR diets to control for differences in food consumption and size to investigate whether the extension in lifespan was due to CR rather than MR. These pair-fed animals had no impairment in growth, meaning the modest reduction in feed intake by the MR animals did not account for the extension in lifespan. Further investigation revealed that MR animals weighed less but consumed more food per gram than their age matched counterparts (i.e., they were slightly hyperphagic).

To identify whether there is genetic variation in the MR response, Zimmerman et al. evaluated MR diets in three diverse strains of rat; Brown Norway, Sprague-Dawley, and Wistar Hannover (18), and demonstrated comparable increases in median lifespan in all of these strains. Miller et al. were the first to report increased lifespan in (BALB/cJ × C57BL/6J) F1 mice on MR diets. In addition, these MR mice displayed lower amounts of plasma Insulin-Like Growth Factor-1 (IGF-1), insulin, glucose, and thyroxine (T4) (19), suggesting novel markers of an endocrine response. These markers were validated in F344 rats fed an MR diet, where MR rats had lower levels of glucose and leptin with corresponding increases in adiponectin, triiodothyronine (T3), and daily energy expenditure when compared to pair-fed controls (20). In the same study, a separate cohort of MR animals were subjected to an oral glucose tolerance test to test the hypothesis that MR preserves insulin action with age. The MR animals responded in a similar fashion to the oral glucose tolerance test as rats fed a control diet at 0, 23, and 72 weeks, although MR animals showed a marked decrease in area under the curve for insulin at both 23 and 72 weeks, demonstrating a preservation of insulin action with age (20). Furthermore, these rats displayed decreased accumulation of visceral fat that, in combination with the endocrine response, supports MR as a strategy to extend lifespan in rodents.

In addition, data support the role of decreased fat deposition, preserved insulin sensitivity, and disruption of the lipogenic/lipolytic balance in adipose tissue as mediators of MRs metabolic phenotype (20–22). This balance was shown to be distrupted by MR through a cycle of increased lipolysis and increased lipogenesis *in vitro* hypothesized to lead to decreases in adipose tissue in older F344 rats (21). In addition, a study found that young and mature animals, as well as obesity prone Osborne–Mendel rats, on a MR diet exhibited long-term increases in energy expenditure and uncoupling protein-1 (UCP-1) in both brown and white adipose tissue. This change in UCP-1 was also accompanied by decreased leptin and increased adiponectin proposing a remodeling of the adipose tissue during MR (23). Authors suggested that MR acts to increase energy expenditure and decrease fat deposition by lowering metabolic efficiency during the night when increases in lipogenesis typically occur (23). These changes explain the extension of healthspan observed in these animals with a complete mechanism for MRs ability to decrease metabolic efficiency (i.e., hyperphagia with decreased growth) (21–24). A recent study detailed that animals introduced to the diet either postweaning or at 80% of mature size had differential effects of hyperphagia (~50 and ~20% increase in energy intake per unit body weight in juvenile and adults, respectively) indicating that the MR diet has differential effects on energy intake and expenditure depending on age (25).

The work detailed thus far has focused on the metabolic effects of MR in mice and rats, which occupies much of the current literature. However, work has also been done in the unique teleost fish model, the rainbow trout; *Oncorhynchus mykiss*. Methionine requirements of these fish are well-known due to their economically important aquaculture status (26–29) and MR has been studied in part due to the implementation of naturally methionine-deficient plant-based diets. In addition, rainbow trout are naturally glucose intolerant making them a potential model for studying the glucose-intolerant phenotype associated with human type-II diabetes and metabolic syndrome (30). In rainbow trout, MR, paired with carbohydrate-enriched diets, was shown to reduce genes associated with *de novo* fatty acid synthesis and reduce circulating plasma glucose 6 h postprandial (30); however, these results must be interpreted cautiously as the design of the diets included no methionine (i.e., methionine deprivation), but adequate cysteine levels. On the other hand, consistently, juvenile rainbow trout fed a formulated MR diet over 6 weeks displayed transcriptional markers of GCN2/eIF2α activation mirroring the effects observed in mammals (31). Conversely, brood stock females fed a similar MR diet for 6 months prior to spawning had reduced triacylglycerol levels with increased total cholesterol and LDL-cholesterol, opposing the cholesterol lowering effects observed in mammals (32). In addition, 48 h of MR *in vitro* in trout hepatocytes resulted in greater levels of glucose uptake due primarily to an increase in the sodium-glucose transporter 2 (33). While the literature is less extensive on the metabolic effects associated with MR in teleosts, it is clear that there is at least a partial conservation of the MR phenotype.

## Physiological Mechanisms of MR

### Lifespan Extension

A proposed mechanism of lifespan extension associated with CR is a decreased rate of mitochondrial reactive oxygen species generation (mitROS) (34). It has been shown that some long-lived species have markedly lower rates of mitROS production compared to their short-lived counterparts (35, 36). Similarly, animals on a MR diet display lower rates of mitROS production and less oxidative damage of mtDNA in both the heart and liver (37). Previous studies have also established that neither carbohydrate restriction nor lipid restriction can replicate the phenotypes seen in CR, PR, and MR (38, 39). This information leads to the hypothesis that changes in mitROS production during both CR and PR could be attributed in part to MR. MR-induced reductions in mitROS production in the heart occurred primarily at complex I, previously established to be the main complex targeted in CR (40). However, in liver MR reduced mitROS in complexes I/III, while CR appears to reduce mitROS only at complex I (41). It should be noted that the studies done on mitROS production during MR had similar effects at both 40 and 80% MR. Since the time of publication, it has been shown that most physiological effects of MR only occur at 80% (42) so the results must be interpreted cautiously.

An additional potential mechanism in the life span response to MR includes an enhanced capacity for a cellular autophagic response and the concomitant acidification of the vacuole (43). Studies done in *Saccharomyces cerevisiae* (Bakers yeast) utilized three different strains of yeast which varied in their ability to synthesize methionine (100% capability, moderate impairment, no *de novo* synthesis). The authors found that lifespan extension was achieved in both the moderate and devoid strains in normal media with the devoid strain demonstrating decreased survival in media with a 90% reduction in methionine. This extension in lifespan was found along with a sharp increase in autophagy followed by acidification of the vacuoles. Additionally, when key genes essential to autophagy were deleted (ATG 5,7,8) this increase in lifespan was ameliorated (43), providing some support for a relationship between autophagic capacity and lifespan, at least in yeast [full review Ref. (44)], although this mechanism has yet to be investigated in MR mammals.

### Metabolic Changes

**Figure 1:**
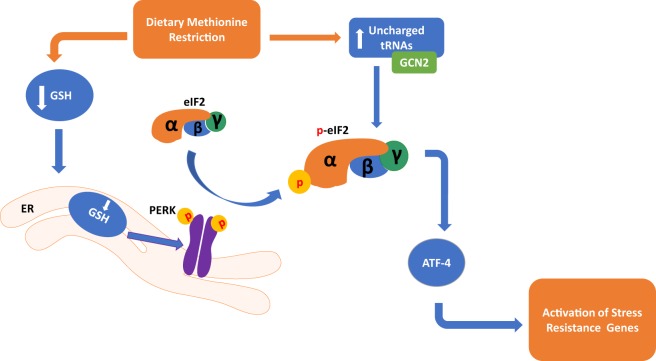
Dietary methionine restriction has been shown to occur through two distinct pathways in the cell. In the endoplasmic reticulum, a reduction of Glutathione (GSH) causes an activation of the PKR-like endoplasmic reticulum kinase (PERK), which activates eukaryotic translation initiation factor 2a (elF2a). In the cytoplasm, a buildup of unchanged tRNAs activates general control non-derepressible 2 (GCN2) which in turn also activates elF2a. Both of these pathways converge in the activation of activating transcription factor 4 (ATF4) and activation of genes that respond to stress.

Two mechanisms related to the sensing of amino acid deficiency may partially explain the metabolic adaptations that take place during MR (Figure 1). A decrease in methionine leads to an increase in uncharged tRNAs that can activate the general control non-derepressible 2 (GCN2) kinase (45, 46), leading to metabolic adaptation during MR. However, GCN2 may not be indispensable for the response as metabolic adaptation can also take place through a noncanonical PKR-like endoplasmic reticulum kinase (PERK)/NRF2 pathway (47). Both of these mechanisms are significant for the MR-induced integrated stress response (ISR) that contributes to cellular homeostasis and regulation of the physiological response. They converge through their mutual regulation of eukaryotic initiation factor-2α (eIF2α) phosphorylation and the subsequent effects on translational capacity *via* eIF2 and synthesis of the active full-length activating transcription factor 4 (ATF4). Active ATF4 is defined as an amino acid sensor (48) and master regulator (49) of metabolism central for controlling expression of genes associated with the stress response, including genes related to lipid metabolism (50), autophagy (51), and maintenance of oxidative stress (52). However, recent evidence indicates that MR restriction can induce ATF4 independent of the eIF2 pathway (53), suggesting that additional mechanisms may be regulating MR-induced ATF4 response.

### Molecular Mechanisms of MR

#### Epigenetics

Nutrigenomics is a rapidly evolving area of research that identifies how nutrition and diet interact with the genome and affect gene expression (54). Epigenetic regulation includes methylation of cytosines within genes or regulatory regions and can activate, but more commonly inhibit, gene expression. Epigenetic regulation also includes histone modifications as methylation, phosphorylation, and acetylation that affect chromatin structure and transcriptional activation. Regulation of methylation status is predominantly controlled by *de novo* methyltransferases and demethylases that acquire their methyl groups from the downstream methionine metabolite, SAM, converting it to *S*-adenosylhomocysteine (SAH).

Age-related changes in the epigenome are implicated in the aging process (55) and although it is established that the epigenome responds to nutrient signals, a direct link between the aging response and nutrient restriction-induced epigenetic effects has yet to be clearly established (56). However, recent evidence indicates that age-related methylation drift, which is hypothesized to be a determinant of mammalian lifespan (57), is attenuated by CR in rhesus monkeys and mice and affects gene expression (58).

Epigenetic effects of protein or MR have largely emphasized consumption during gestation and subsequent effects on offspring development. This concept of “nutritional programming” actually supports dietary supplementation of methyl donors like folate, choline, and methionine to improve offspring health and growth potential, and the extent to which this response is regulated by differential methylation of genes critical for development remains unclear (59). There is, however, a small body of work examining the epigenetic response to MR as it relates to aging. *In vitro* findings indicate that physiologically relevant concentrations of methionine in cell culture media alter the SAM/SAH ratio and methylation status of H3K4trimethyl, leading to regulation of gene transcription (60). The methionine levels in the serum of humans exhibit enough variation during MR to alter histone methylation (60). Similar findings were observed in adult (but not young) mice in which short-term (12 weeks) MR (0.12%-MR, 0.84%-CD) decreased SAH concentration and increased global DNA methylation in liver, although the opposite was observed in adipose tissue (61). These studies collectively support a role for changes in epigenetic marks by MR, although additional research is needed to further characterize the gene-specific responses.

#### MicroRNAs

MicroRNAs are small endogenous 18–22 base pair nucleotide sequences (62) that play roles in mediating posttranscriptional gene silencing in mammals, plants, and invertebrate organisms (63, 64). They are synthesized from primary miRNAs by two enzymes Drosha in the nucleus and Dicer in the cytoplasm (65) and are bound by Argonaute subfamily of proteins before integration into the RISC complex. After integration miRNAs have the ability to post transcriptionally regulate gene expression through the formation of RNA duplexes (62). The miRNA profile changes with age and numerous miRNAs that are affected by age are also regulated by CR (66), suggesting these regulatory RNA molecules are associated with CR-induced extension of lifespan. Many of these miRNAs associated with the IGF-1 PI3K/AKT mTOR signaling pathways are important for nutrient sensing and subsequent physiological response during CR.

Recent evidence also supports a role for miRNAs in the MR phenotype in teleosts and mammals. Rainbow trout myosatellite cells exposed to methionine-deficient cell media regulate miRNAs that reduce their capacity for differentiation (miR-133a, miR-206, and miR-210) (4). Rainbow trout fed an MR (0.775%) diet for 4 weeks had lower levels of miR-133a in the skeletal muscle at 4 weeks and increased glucose tolerance following a glucose challenge at 8 weeks (5). Mice consuming an MR diet (0.12%) exhibit increased expression of miR-133a, miR-335-5p, and miR-204 in the bone marrow as well as miR-31 in the plasma and liver (5, 6, 67, 68). Although these studies did not directly investigate the aging response, they do indicate that miRNAs are regulated by methionine availability and potentially play a role in the metabolic phenotype.

While miRNAs have traditionally been known to repress transcription, evidence suggests that cells undergoing an ISR, such as those under MR, may activate miRNAs to regulate a unique suite of mRNAs to maintain homeostasis (69). As previously mentioned, a potential mechanism of MR is through the GCN2 amino acid deprivation pathway and the PERK/NRF2 (Figure 1) pathway. Both of these kinases phosphorylate eIF2α, which activates ATF4 and induces the formation of stress granules (70) containing miRNAs, mRNA targets, and Argonaute proteins (71). Similar to MR, leucine deficiency activates GCN2/ATF4 (72), which increases expression of miRNA-212-5p that subsequently reduces lipid accumulation (72) and enhances gluconeogenesis (73). Although the effect of leucine restriction on lifespan is opposite that of MR, activation of GCN2/ATF4 and subsequent regulation of specific miRNAs appears to be a common response to cellular stress induced by amino acid restriction.

While the notion of miRNA involvement in the MR phenotype is new, it is well-known that a few miRNAs regulate cellular stress response in other situations. miR-211 is known as a pro-survival miRNA during ER stress activated by the PERK pathway (74). In this circumstance, miR-211 is used to attenuate the amount of the pro-apoptotic transcription factor *chop* and keep the cell from initiating apoptosis during periods of short-term ER stress. miR-122 causes a repression of the cationic amino acid transporter-1 under normal conditions. Under varying conditions of stress, this miR can de-repress the mRNA transcript and cause it to be localized to the polysomes (75). For a full review of how miRNAs are regulated during stress responses, see Leung and Sharp (76). Because MR is known to activate the PERK pathway without induction of ER stress (47) the connection between MR and miRNAs must be interpreted carefully and warrants further study.

## Conclusion and Future Perspectives

**Figure 2:**
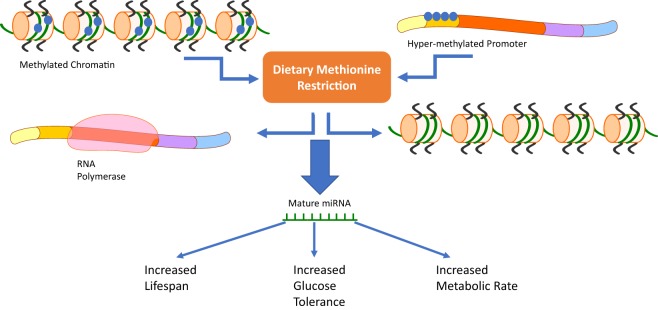
Our review suggests that a third pathway should be added to the already known mechanisms of methionine restriction (MR). Changes in chromatin methylation or methylation status of DNA promoters due to dietary MR has the ability to activate or repress microRNAs (miRNAs) involved in the cells response to MR. These distinct changes could then be related to MRs phenotype characterized by increased lifespan, glucose tolerance, and metabolic rate.

The evidence presented clearly shows MRs role in altering metabolic phenotypes in both mammals and teleosts. The activation of miRNAs during MR provides a potential link between changes in methylation and the ISRs in cells. Studies utilizing rainbow trout myosatellite cells *in vitro* and juvenile rainbow trout *in vivo* (4, 5) have shown that methionine can regulate the level of expression of miRNAs in teleosts (Figure 2). Studies done in mammalian systems have also shown that miRNAs are differentially regulated in the plasma, liver, and bone marrow of MR mice (6). This review explores mechanisms responsible for the MR phenotype including miRNAs, amino acid starvation, and stress response pathways.

Future research should investigate miRNAs in circulation (77) during MR. miRNAs found to be altered during MR (78) can then be analyzed for their role in controlling muscle-specific transcription factors like MyoD and myogenin (79) and can also be explored in other tissues to observe conservation of function during stress responses in tissue (80). Indeed, the master regulator of many of these mechanisms may lie in epigenetic changes that occur during MR. These changes have been hinted at in previous literature (60, 61) but have not yet been fully explored.

## Author Contributions

ML: writing, reviewing, and conception. KF: writing. BC: writing and reviewing. PB: writing, reviewing, and conception.

## Conflict of Interest Statement

The authors declare that the research was conducted in the absence of any commercial or financial relationships that could be construed as a potential conflict of interest.
